# Factors Influencing Manipulation of a Familiar Object in Patients With Limb Apraxia After Stroke

**DOI:** 10.3389/fnhum.2019.00465

**Published:** 2020-02-11

**Authors:** Gloria Pizzamiglio, Zuo Zhang, Mihaela Duta, Elisabeth Rounis

**Affiliations:** ^1^Wellcome Centre for Human Neuroimaging, Institute of Neurology, University College London, London, United Kingdom; ^2^Nuffield Department of Clinical Neurosciences, University of Oxford, Oxford, United Kingdom; ^3^Social, Genetic and Developmental Psychiatry Centre, Institute of Psychiatry, Psychology and Neuroscience, King’s College London, London, United Kingdom; ^4^Department of Experimental Psychology, University of Oxford, Oxford, United Kingdom

**Keywords:** apraxia, goal-directed actions, habitual actions, affordances, object manipulation

## Abstract

Previous studies have shown that hand actions to visual objects are affected both by perceptual factors and by action goals. Our aim was to study how these processes affected hand actions in chronic stroke patients, based on whether they had limb apraxia. Twenty-two left hemisphere, chronic stroke patients were measured on neuropsychological tasks of limb apraxia, which was identified in a subgroup of 10 patients. All patients underwent testing on a separate task of making simple reach and grasp actions to a cup. Their performance was compared to a group of 18 healthy age-matched volunteers. Participants were instructed to grasp the top or bottom of a cup to either lift or turn it over so as to end with a hand position that was either comfortable or uncomfortable. This task tested the influence of the compatibility of hand–cup orientation, as well as goals driven by the end-state comfort of the hand, on action selection for object manipulation. Participants’ performance was measured in terms of error rates, and speed of initiation and reaching (movement time) to the object. The patients’ performance was significantly delayed, and error rates increased when reaching to grasp a cup under conditions of poor compatibility and end-state comfort. The subgroup of patients with apraxia showed a decreased influence of compatibility of hand interaction with the cup, with increased error rates and delayed response times, compared to patients with no apraxia and healthy volunteers. This is despite the fact they did not display significant deficits on neuropsychological tasks of real object use. The study shows that patients with apraxia have difficulties in selecting elements of object-directed actions, pertaining to both habitual and goal-directed factors.

## Introduction

A large number of movements can be used to achieve a goal, such as grasping to move an object. However, studies have demonstrated that skilled actions, such as object manipulation, are often stereotyped (Keele, 1968; Harris and Wolpert, 1998).

Two main factors driving object manipulation include features relating to the object’s and the environment’s properties [such as its shape, position, and size (Jeannerod, 1994)], as well as what one intends to do with the object, namely, action “goals” (Marteniuk et al., 1987; Rosenbaum et al., 2006). The latter rely on an evaluation of expected outcomes (Rosenbaum et al., 2006; Grafton and Hamilton, 2007).

Most studies examining the perceptual effects of object properties on hand actions have identified stimulus–response compatibility effects. Gibson (1979) introduced the concept of “affordance.” This described how visual properties of objects, or the environment, can give rise to action representations, depending on contextual demands of the task. “Affordances” link graspable features of an object and an independent action elicited in a task. In a seminal study, participants responded faster if the orientation of the handle on an object was compatible with the hand used to respond, in a task in which they had to make right- or left-finger presses according to whether objects in pictures were depicted as upright or inverted (Tucker and Ellis, 1998). This is despite the fact that they were not required to make a judgment about the handle orientation. Other studies have replicated this effect (Ellis and Tucker, 2000; Bub and Masson, 2010).

The effect of action outcomes on object manipulations has been described using a phenomenon named “end-state comfort” effect (Rosenbaum et al., 1990, 1992). This reflects the preference for participants to select uncomfortable grip postures at the start of an action toward an object, in order to end in a comfortable posture. This effect most likely reflects a bias on the choice of one action in the face of an overwhelming number of possible others, when reaching to grasp an object (Wolpert and Miall, 1996).

Although previously these two factors were studied in isolation, recent investigations have looked at both combined (Herbort and Butz, 2011, 2015; Herbort et al., 2017; Rounis et al., 2017). Herbort and Butz (2011) and Rounis et al. (2017) investigated the effect of turning a cup either with the thumb positioned toward its top (lip) or with a thumb positioned in the opposite direction. Both studies identified an ‘End-state comfort’ effect indicating a preference for turns that started with an inverted (or pronated) grasp to end comfortably (in a supinated) grasp. However, this effect was mitigated by an “affordance” effect measured either in the choice of grasping the cup from its lip, even though it would result in an uncomfortable end state, for example, when the cup was upright in the Herbort and Butz (2011) study, or in mitigating the end-state comfort effect measured using response and movement times (MTs) in “afforded” actions (Rounis et al., 2017). Further studies have demonstrated the effect of affordances trumping the end-state comfort effects, suggesting that habitual actions provided by the former exert a separate influence on goal-directed planning (Herbort et al., 2017).

Limb apraxia is a disorder of skilled action that does not result from motor weakness, incoordination, incomprehension, or sensory impairment, following an acquired brain lesion, such as a stroke. Traditional theories of the disorder distinguish between “ideational” and “ideomotor” apraxia. In the former, patients lose the ability to represent an action conceptually and display difficulties in knowing how to use an object, despite being able to name it and knowing its function. In the latter, more common condition, actions are conceptually correct but implemented poorly. Patients with ideomotor apraxia are unable to imitate meaningless gestures and show spatiotemporal errors in pantomiming or using objects (Sirigu et al., 1995; Buxbaum et al., 2003).

Previous investigations of patients with ideomotor apraxia have hypothesized deficits in selecting among competing actions relating to “affordances” (Buxbaum et al., 2003; Jax and Buxbaum, 2013; Watson and Buxbaum, 2014, 2015; Rounis and Humphreys, 2015). This is further supported by neuropsychological findings in which patients often overrely on affordances at the expense of goal-directed behavior (Riddoch et al., 1989; Osiurak et al., 2008a, b).

We developed a new experimental procedure to assess the interplay between the compatibility of object features and goal-directed influence in the form of hand posture preferences at the end of the action, during motor planning using the task described in Rounis et al. (2017). We varied the compatibility of the initial start posture of the hand with the physical properties of a target object and the preferred end posture for the action (whether it was comfortable or uncomfortable). A group of patients with and without apraxia, and a group of healthy age-matched participants were instructed to either lift or turn a cup presented in front of them by grasping it from a specified position (namely, its lip, which corresponded to the open end of the object that could be filled, or its bottom, which corresponded to the closed end). The cup itself was placed in an upright orientation in one half of the trials or upside down in the other half. This led to four possible actions: lift with a supinated grasp or lift with a pronated grasp and turn with a supinated grasp, ending in a pronated (uncomfortable) posture, or turn with a pronated grasp, ending with a supinated (comfortable) posture.

We hypothesized that there would be dissociable effects of (i) an initial grasp preference, or “compatibility,” and (ii) an end-state comfort effect, related to whether the posture of the hand at the end of the task was comfortable or not, in healthy volunteers and patients with and without apraxia. This would be demonstrated if patients with and without apraxia displayed differences in the compatibility and end-state comfort effects compared to healthy volunteers. Previous literature would suggest that patients with apraxia would be impaired in actions that were not compatible (Osiurak et al., 2008b; Lee et al., 2014).

## Materials and Methods

### Participants

Twenty-two, left hemisphere chronic (>1 year) stroke patients (16 males, 6 females) aged between 25 and 79 years (mean age, 56.6 years) took part in the study. They had suffered their first ever stroke more than 1 year ago (mean time since stroke: 20.4 months). Details of the patient demographics are provided in Table 1.

**TABLE 1:** Patient demographics.

Patient no.	Handedness/hand used	Education years	Months since stoke	ARAT	Imitation	GP	GR
1	R/R	13	27	100	100	100	100
2	R/R	13	14	100	100	100	100
**3**	**R/R**	**11**	**22**	**93**	**70**	**58.3**	**100**
4	R/R	12	16	93	90	100	100
5	R/L	12	13	0	100	91.7	100
6	R/R	17	21	100	95	92	100
7	R/R	12	16	100	100	100	100
8	R/L	11	23	0	85	91.7	100
**9**	**R/R**	**15**	**17**	**96.5**	**35**	**8.3**	**83.3**
**10**	**R/L**	**15**	**21**	**73.7**	**70**	**58.3**	**83.3**
11	R/R	19	14	100	100	100	100
12	R/L	12	16	86	90	83.3	100
13	R/L	16	28	89	85	100	100
14	R/R	13	27	100	90	83.3	100
**15**	**R/L**	**14**	**16**	**0**	**70**	**50**	**66.7**
16	R/R	13	30	100	100	100	100
**17**	**R/L**	**11**	**21**	**83**	**70**	**75**	**100**
**18**	**R/L**	**13**	**25**	**0**	**70**	**75**	**83.3**
**19**	**R/L**	**11**	**15**	**0**	**70**	**75**	**100**
**20**	**R/R**	**11**	**18**	**96.5**	**65**	**66.7**	**100**
**21**	**R/L**	**15**	**17**	**0**	**45**	**75**	**100**
**22**	**R/L**	**13**	**32**	**0**	**70**	**75**	**83.3**

*Patients with ideomotor apraxia have been highlighted in bold. R = Right hand, L = Left hand, and All scores were normalized out of 100.*

An additional 18 healthy volunteers (10 males, 8 females) aged between 24 and 77 years (mean age, 58.9 years) with no history of any neurological and psychiatric illnesses took part in the study.

Written informed consent was obtained from all participants taking part in the study, which was approved by the Health Research Authority, South Central – Berkshire Ethics Committee.

Participants attended the Cognitive Neuropsychology Centre at the Department of Psychology, University of Oxford, for two separate sessions. In the first session, they underwent detailed neuropsychological testing of apraxia and cognitive function. In the second session, they undertook a behavioral task, involving cup manipulation.

All participants were right handed (Oldfield, 1971). Patients used their dominant hand or, if they had hemiparesis, their unaffected hand, to complete the task. A total of 11 out of 22 patients used their left hand due to hemiparesis. The remaining patients and healthy volunteers used their right hand for the performance of all tasks in this study. The degree of impairment caused by stroke was formally assessed using the Action Research Arm Test (ARAT) scale (Lyle, 1981).

### Neuropsychological Testing

Participants taking part in the study underwent cognitive screening and assessment of praxis deficits. We report the neuropsychological data of praxis deficits from screening using three tasks, which were derived from the Birmingham Cognitive screen. These comprised a meaningless gesture imitation task, a gesture production, and a gesture recognition task.

#### Meaningless Gesture Imitation

The meaningless gesture imitation task was derived from the apraxia testing section of the Birmingham Cognitive Screening tool, version 2 (Humphreys et al., 2012). The task required imitation of 10 non-symbolic gestures using the subject’s least paretic hand (see above), all of which required holding a static position after demonstration by the experimenter. The test consisted of four gestures involving whole-hand movements and six involving independent finger movements. They were performed slowly by the experimenter in front of the participants for them to reproduce immediately afterward. If an item was not reproduced flawlessly on the first presentation, a second trial was given. The participants’ performance was video-recorded and assessed by two separate assessors.

Patients scored 2, 1, and 0 depending on whether their imitation was correct on the first or second presentations or never succeeded. This led to a scoring out of 8 for the hand gestures and out of 12 for the finger gestures, with the total score being out of 20.

#### Gesture Production

The gesture production task involved pantomime of a total of six gestures (three transitive, involving pantomime of object use, e.g., “show me how you would brush your teeth, using a toothbrush in your hand”; three intransitive, involving pantomime of a familiar gesture to verbal command that does not require object use, e.g., “show me how you stop traffic”). The test included body-centered (salute, using a glass), non-body-centered (stop, using a salt cellar), repetitive (hitch hiking, using a hammer), and non-repetitive (stop, using a glass) actions. All actions can be carried out as a single step sequence. Patients were allowed a maximum of 15 s per item to respond and were asked to execute the action once. Two points were given for a correct and accurate gesture; 1 point was given for a recognizable but inaccurate gesture (e.g., including spatial and/or movement errors); and 0 points were given for no response after 15 s, an unrecognizable response, or perseveration from previous gestures. The final sum score (maximum, 12) was used in the analyses.

#### Gesture Recognition

In the gesture recognition task, the examiner produced six actions that patients had to recognize: three transitive (using a cup, using a key, using a lighter) and three intransitive (come over, good, goodbye) actions. The examiner showed each gesture while the patients had to select the action being performed from a multiple-choice list, which included four alternative responses for each action. The four alternatives for each action corresponded to: (1) the correct action (e.g., using a lighter), (2) a semantically related action (using a match), (3) a visually related action (using a gun), and (4) an unrelated action (using a torch). The patients were allowed a maximum of 15 s per item to respond. and they were given 1 point for each correct response. The final sum score (maximum, 6) was used in the analyses.

The data from both transitive and intransitive gestures in these tasks were entered together as a composite measure.

#### Single-Object Use

The single-object use task was aimed at identifying patients with ideational apraxia. Patients were presented with one of six objects individually, one at a time (a torch, a straw, a comb, a nail clipper, a screwdriver, and matches). They were asked to demonstrate the use of each of these with the object at hand. The patients were allowed a maximum of 15 s per item to respond. Two points were given for a correct and accurate gesture; 1 point was given for a recognizable but inaccurate gesture (e.g., including spatial and/or movement errors); and 0 points were given for no response after 15 s, an unrecognizable response, or perseveration from previous gestures. The final sum score (maximum, 12) was used in the analyses. Of note as there were no normative data to establish cutoff scores, we compared the performances of patients and healthy volunteers on this task using a paired-sample *t*-test (assuming unequal variance).

Table 2 shows the praxis task cutoff scores for tasks of ideomotor apraxia from the Birmingham Cognitive Screening program, based on fifth percentile across age groups in percent estimates of the score (from Humphreys et al., 2012). Patients were characterized as having ideomotor apraxia if they scored below any of the cutoff scores of gesture production and the imitation of meaningless gesture tasks (Buxbaum, 2001; Buxbaum et al., 2003). Patients’ hand gestures were videotaped and saved anonymously. They were scored offline by two independent assessors (ER and GP). None of the stroke patients had a formal diagnosis of ideomotor apraxia known to the assessors.

**TABLE 2:** Praxis task cutoff scores, according to age (Humphreys et al., 2012).

	Age range (*Number of controls tested*)		
	≤64	65–74	≥75
	*(N* = *34)*	*(N* = *33)*	*(N* = *33)*
Gesture production	83%	83%	83%
Gesture recognition	83%	83%	67%
Gesture imitation	75%	75%	75%

### Other Comparisons

We performed additional comparisons by correlating apraxic deficits with motor impairment measures on the ARAT score of the paretic hand (see Table 1), as well as with constructional apraxia using the Rey-Osterrieth figure copy test (Rey, 1941; Osterrieth, 1944) looking for any presence of visuospatial deficits in patients. Correlation analyses used Spearmann’s rho and Bonferroni correction for multiple comparisons with a corrected *p* < 0.05 considered as significant.

### Behavioral Study: Object Manipulation Task

To assess the interplay between the compatibility of object features and hand posture preferences during motor planning, we used a new experimental procedure. This has been described in a previous study (Rounis et al., 2017).

This task involves the manipulation of a familiar object, namely, a cup, in which we varied the compatibility of the initial start posture of the hand with the orientation of the object. The former manipulation was indicated by specifying the position to be grasped on the cup and determined the preferred end posture for the action (whether it was comfortable, usually when ending in a supinated position, or uncomfortable, when ending in a pronated position). The latter manipulation determined the physical properties of our target object by presenting the cup in its upright position, which is favorable for object use as the open end of the cup was “up,” or upside-down position. The goal of the action, provided with a verbal instruction, was to either lift or turn the cup, which resulted in different action outcomes, based on the end-state comfort effect.

#### Materials

**FIGURE 1:**
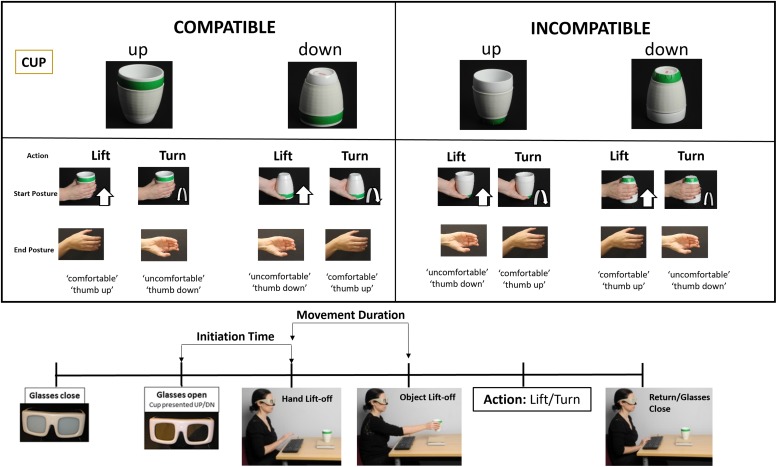
Conditions and task. The **top panel** shows the eight task conditions. Participants were asked to “lift” or “turn” a cup by grasping it from the green line marking. In half of the trials, this marking was compatible with the familiar way of grasping a cup (with the thumb pointing toward its open end), and in the other half, it was in the opposite (closed end) of the cup. The end-state comfort depended on the initial hand position and action performed. It was either comfortable (meaning that the thumb was oriented up at the end of the action) or uncomfortable (thumb oriented down). The **bottom panel** shows the time course of a trial. Participants started with their hand resting on a keyboard. The initiation times represented the time between opening of the glasses, which coincided with a verbal cue indicating the action (to lift or turn the cup), and the time of lift-off of the hand from its resting position on the keyboard. The movement durations represented the time between lift-off of the hand and the time to lift the object from its pad, to carry out the action. This represented the time reaching to grasp the cup. *Please note that the individuals present in the figure gave informed consent to have this image published.*

A cup with no handle (“bodum” cup; Figure 1) was used as the target object in this experiment. The cup dimensions were as follows: 50 mm wide at its base, 98 mm wide at its top, and 118 mm in height, and it weighed 270 g. The center of mass of the cup was located 9.8 cm from its base. It was placed at the center of a wooden platform measuring 20 × 10 cm that sat on top of a cedrus response box 30 cm in front of the participants. On 50% of the trials, the cup was upright, and in the remaining 50%, it was oriented upside down.

#### Task

Participants used their dominant (right) hand to perform this task. In the case of hemiparesis, patients used their non-dominant hand. A total of 11 patients used their left hand to complete the task. Their hand rested on a keyboard at baseline, between trials. A trial started with the opening of liquid crystal “PLATO” spectacles (Translucent Technologies, Toronto, ON, Canada). This allowed for the timely visualization of the object on the pad. A simultaneous verbal auditory instruction triggered from the computer, lasting 1 s indicated the action to be performed on the cup. The action was either to “lift” or to “turn” the cup (50% of the trials were allocated for each instruction, respectively).

A green horizontal line on the cup specified the *initial* hand posture to be used. If the line was at the top, participants had to grasp the cup using a supinated wrist posture, with their thumb facing “up.” If the line was placed at the bottom, participants were instructed to grasp the cup using a pronated wrist posture, with their thumb facing “down.” In each case, participants were asked to align their thumb and forefinger with the line. These grasps were made independent of the cup orientation. Hence, in 50% of the trials, the initial wrist posture was congruent with the cup orientation (if the line was on the same side as the open end of the cup), and this was the case whether the cup was in its upright position (in which case, the line was on the side of the open end of the cup and the grip was supinated) or upside down (in which case, the grip was pronated). We carefully designed our cup selection task so that stimuli were presented vertically rather than horizontally (we used a cup with no handles). This was to prevent a possible confound of visuospatial attention. Previous studies have used central stimuli to distinguish motor attention from visuospatial attention (Rounis et al., 2007). The two are separable both in terms of their anatomical localization (motor attention is lateralized to the left and centered in the supramarginal gyrus) and in terms of its function in orienting attention in a limb-centered representation of space (Rushworth et al., 2001; Rounis et al., 2007; Rinne et al., 2018).

The action, either to lift or turn the cup, determined the final end posture, which was again either pronated or supinated depending on the *initial* grip instruction and the action.

When the action was completed, participants returned their hand to the resting position, which led to the closure of the PLATO spectacles. Performance of this task was under full direct vision, and the spectacles only closed after completion of the action and upon return to the resting position. They therefore remained open for the action for an average of 4 s (±0.5 s). Participants were asked to complete their action as quickly and as accurately as possible.

The experiment was programmed on Matlab 2014b using Psychtoolbox version 3.0, triggered from a Windows PC. The experimenter recorded errors or adjustments in the grasp position and changed the cup condition for the next trial. The movements performed on each individual trial were video-recorded and reviewed offline, to assess the movement accuracy. *Post hoc* analyses of the movements revealed that the cups were lifted on average 10.5 cm from the platform for “lift” actions and 10.7 cm from the platform for “turn” actions.

All trial types (grasp top or bottom of the cup, lift or turn) were presented in a pseudorandom order in a total of 17 miniblocks, to ensure that all trial conditions were repeated the same number of times, for averaging. Each miniblock consisted of one trial from each of the eight trial conditions. The first set of miniblocks was always eliminated from the analysis. The total number of trials per session was 136, of which the first 8 were discarded, so only 128 were analyzed for each participant.

This arrangement allowed us to measure response times at two time points, reflecting movement preparation for the action performed in this task. The *initiation time* measured the time between the stimulus onset (corresponding to the opening of “PLATO” goggles allowing viewing of the cup and verbal instruction) and the time at which the participants lifted their hand from the resting position on the spacebar to initiate an action toward the cup. The *movement duration* was the time between the release of the spacebar and the lift of the object from the platform, measured by the trigger of buttons from the cedrus box on which the platform was positioned (Figure 1, bottom panel).

The choice of these timings was based on results from our previous study in Rounis et al. (2017), in which we observed different effects of affordance and end-state comfort. The initiation times represented planning during which participants likely make a decision about which action to perform and how to implement it (Welford, 1968; Wong et al., 2015). The MT measured the reach to grasp the object, reflecting effector-based movement implementation (Cisek, 2005; Bub and Masson, 2010).

#### Data Analysis

Any errors were recorded by the experimenter using a graphical user interface. In addition, correct response latencies lower than 200 ms or longer than 3500 ms for initiation times were excluded as outliers. The lower and upper bounds chosen for these time points were set so that no more than 0.5% of correct responses were excluded either due to being classified as a false start or as an unusually prolonged response [see Ulrich and Miller (1994); Bub and Masson (2010) for a similar approach].

We hypothesized that actions with the initial grasp oriented toward its open end (or its lip) would be faster than a grasp to the closed end of the cup, as observed in our previous study (Rounis et al., 2017). For an upright cup, this would correspond to a supinated grasp; and for a cup oriented down, a pronated grasp (Figure 1). This concurs with previous studies that have shown that participants are more likely to choose a hand orientation appropriate for the object’s use (Creem and Proffitt, 2001; Herbort and Butz, 2011).

The correct responses for errors and for each time point (initiation and movement durations) were submitted to separate analyses of variance (ANOVAs) using IBM SPSS Statistic 22 for Windows software (SPSS Inc., Chicago, IL, United States). The type I error rate was set at 0.05 for the analyses reported here. Greenhouse–Geisser correction for degrees of freedom was used when assumption of sphericity was not met. Interaction effects were evaluated with paired *t*-tests (*p* < 0.05 or *p* = 0.05), assuming equal variance within group and unequal variance for between-group comparisons.

A mixed-model, nested ANOVA was used to identify between-subject effects of GROUP (with three levels: 10 left hemisphere patients with, 12 without apraxia, and 18 healthy volunteers), investigating the three within-subject factors (with two levels each), as reported in our previous study (Rounis et al., 2017). These included COMPATIBILITY (previously referred to as an initial grasp preference (or “affordance”) for positioning the thumb toward the cup’s open end), ACTION (which determined whether the task was to “lift” or to “turn” the cup), and the END-STATE COMFORT of the hand after the action is completed [the end state being comfortable (“thumb up”) or not (“thumb down”)]. Figure 1 shows the task conditions and experimental setup. Table 3 outlines the behavioral results on this study for each patient category and task conditions, summarized in terms of compatibility and end state comfort effects.

**TABLE 3:** Results for error rates, and initiation and movement times, in compatible and incompatible trials, and in trials that ended comfortably versus the ones that did not.

	Healthy volunteers (18 participants)		Patients without apraxia (12 participants)		Patients with apraxia (10 participants)	
Number of errors (COMPATIBILITY)	Compatible: Mean: 1.4 (SEM = 0.37)	Incompatible: Mean: 2.5 (SEM = 0.53)	Compatible: Mean: 7 (SEM = 2.3)	Incompatible: Mean: 9 (SEM = 2.8)	Compatible: Mean: 12 (SEM = 4)	Incompatible: Mean: 19 (SEM = 5)
Number of errors (END-STATE COMFORT)	Comfortable Mean: 0.78 (SEM = 0.15)	Uncomfortable Mean: 3.1 (SEM = 0.53)	Comfortable Mean: 9 (SEM = 1)	Uncomfortable Mean: 39 (SEM = 5)	Comfortable Mean: 2 (SEM = 0.8)	Uncomfortable Mean: 6 (SEM = 1.6)
Initiation times in ms (COMPATIBILITY)	Mean: 838.60 (SEM = 45.80)	Mean: 873.42 (SEM = 48.08)	Mean: 913.17 (SEM = 117.29)	Mean: 920.61 (SEM = 118.25)	Mean: 1057.44 (SEM = 92.25)	Mean: 1106.79 (SEM = 108.71)
Initiation times in ms (END-STATE COMFORT)	Mean: 837.52 (SEM = 44.74)	Mean: 874.51 (SEM = 45.18)	Mean: 898.20 (SEM = 110.79)	Mean: 935.98 (SEM = 125.62)	Mean: 1034.53 (SEM = 77.73)	Mean: 1189.25 (SEM = 121.55)
Movement times in ms (COMPATIBILITY)	Mean: 785.8967 (SEM = 30.12)	Mean: 828.4889 (SEM = 29.74)	Mean: 1036.02 (SEM = 92.33)	Mean: 1086.03 (SEM = 92.43)	Mean: 1629.45 (SEM = 203.41)	Mean: 1877.20 (SEM = 276.92)
Movement times in ms (END-STATE COMFORT)	Mean: 775.08 (SEM = 29.94)	Mean: 826.07 (SEM = 33.42)	Mean: 1008.13 (SEM = 87.80)	Mean: 1113.93 (SEM = 106.06)	Mean: 1658.39 (SEM = 216.64)	Mean: 1848.26 (SEM = 259.77)

*Ms, milliseconds, SEM, standard error of the mean.*

## Results

### Neuropsychological Tasks

A total of 10 out of 22 left hemisphere stroke patients performed below the cutoff scores on assessments of gesture production and meaningless gesture imitation, indicating ideomotor apraxia. There was a complete agreement between the raters in defining those patients who had ideomotor apraxia (Cohen’s kappa = 1). The patient results are reported in Table 1.

Performance on the single-object use task revealed no significant differences between patients (average score = 11.64, SEM = 0.16) and healthy volunteers (average score = 11.94, SEM = 0.05, *t* = -1.8, *p* = 0.08).

We found that the level of stroke impairment on the hemiparetic hand measured with ARAT significantly correlated with gesture production (rho_22_ = 0.520, *p* = 0.004), meaningless gesture imitation (rho_22_ = 0.556, *p* = 0.002), and the gesture recognition task (rho_22_ = 0.416, *p* = 0.018).

Comparisons between results in the Rey-Osterrieth figure copy task (subdivided in total score as well as subscores for left-, middle-, and right-sided copies) and each of the praxis tasks revealed no significant correlations between the two (*p* > 0.1).

### Behavioral Task

#### Error Rates

Healthy volunteers made a total of 3.03% errors, and stroke patients made a total of 17.86% errors. Error trials were discarded from reaction time and MT analyses (70 out of 2304 trials in healthy volunteers, 503 out of 2816 in stroke patients). Of note, a review of error types identified that most errors related to incorrect or adjustments in grasp [with <0.04% of errors relating to other factors such as incorrect initiation or action (lifting instead of turning)]. Due to insufficient numbers in categories other than grasp errors, errors were combined and analyzed according to trial condition rather than error type.

Error rates, and initiation time and MT were submitted into three separate mixed-model nested ANOVA, with a between-subject factor of GROUP (three levels: healthy volunteers and left hemisphere stroke patients WITH and WITHOUT apraxia) and within-subject factors of COMPATIBILITY (two levels: with the initial hand posture matching the object orientation, with the thumb orientation toward the lip of the cup), ACTION (two levels: lift versus turn), and END-STATE COMFORT (two levels: starting with a pronated to end in a supinated, comfortable grasp, or starting with a supinated grasp to end in a pronated, uncomfortable one).

**FIGURE 2:**
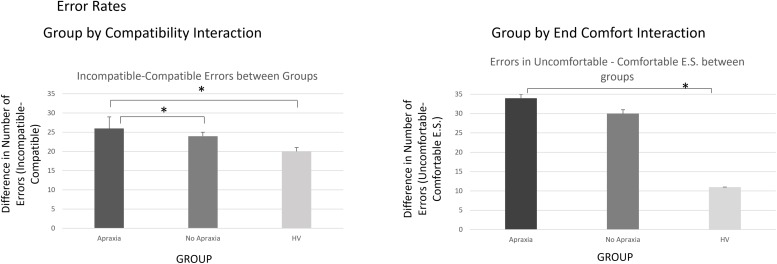
Error rates – interactions by group. The **left panel** demonstrates the compatibility by group interaction, indicating that the error difference for incompatible trials was more significant in patients compared to healthy volunteers, particularly if they suffered from ideomotor apraxia. The **right panel** demonstrates the end-state comfort effect on error rate differences between groups, which were significantly more elevated for patients, particularly those with ideomotor apraxia, compared to healthy volunteers. ^∗^*p* < 0.05.

The analysis on error rates revealed the main effects of COMPATIBILITY [*F*(1,37) = 17.2, h^2^ = 0.32, *p* < 0.0001], with on average 2.75 more errors on incompatible than on compatible trials. There was also a significant effect of END-STATE COMFORT [*F*(1,37) = 26.1, h^2^ = 0.41, *p* < 0.0001], with on average 7.35 more errors on trials that ended uncomfortably than those ending comfortably. The main effect of ACTION was not significant [*F*(1,37) = 0.15, h^2^ = 0.004, *p* = 0.7]. These significant main effects interacted with the between-subject factor of GROUP, leading to significant GROUP by COMPATIBILITY [*F*(1,2) = 4.35, h^2^ = 0.19, *p* = 0.02] and GROUP by END-STATE COMFORT [*F*(1,2) = 4.28, h^2^ = 0.19, *p* = 0.02]) effects. The results of *post hoc* analyses describing these effects are outlined in the Supplementary Material and in Figure 2.

#### Initiation Times

The analyses on initiation times revealed the significant main effects of COMPATIBILITY [*F*(1,37) = 7.03, h^2^ = 0.16, *p* = 0.01], indicating that compatible trials were initiated 30.5 ms (SEM = 2.9 ms) more rapidly than incompatible trials. The main effects of ACTION [*F*(1,37) = 10.12, h^2^ = 0.21, *p* = 0.003] and END-STATE COMFORT [*F*(1,37) = 19.02, h^2^ = 0.34, *p* < 0.0001] were also significant. Lift actions were initiated faster by an average of 64.5 ms (SEM = 2.5 ms) than turn actions, and initiation times for actions that ended comfortably were 63.25 ms (SEM = 10.35 ms) shorter than for actions that ended uncomfortably. There were two-way interactions of GROUP BY END-STATE COMFORT [*F*(2,37) = 3.99, h^2^ = 0.18, *p* = 0.03] and ACTION BY END-STATE COMFORT [*F*(1,37) = 7.04, h^2^ = 0.32, *p* = 0.01].

**FIGURE 3:**
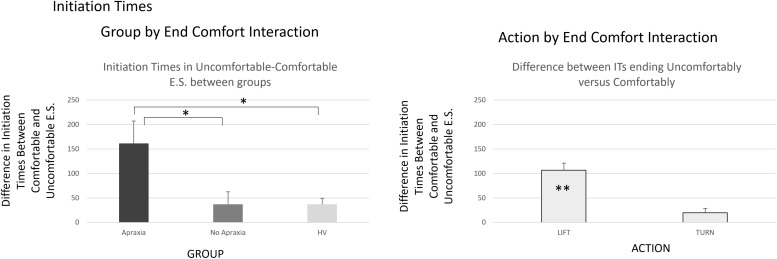
Initiation times – interactions by group. The group by end-comfort interaction in the **left panel** indicated that initiation times for actions that ended uncomfortably were longer than those for the ones that ended comfortably, particularly in patients with ideomotor apraxia, compared to patients without apraxia and healthy volunteers. The action by end-state comfort interaction indicated in the **right panel** was due to prolonged initiation times for actions ending uncomfortably in lift actions, when there was no significant difference in initiation times for turn actions whether they ended comfortably or not. ^∗^*p* < 0.05, ^∗∗^*p* < 0.001.

The results from our *post hoc* analyses appear in the Supplementary Material and are shown in Figure 3.

#### Movement Times

This analysis revealed the main effects of COMPATIBILITY [*F*(1,37) = 8.00, h^2^ = 0.18, *p* = 0.008], indicating that MTs for trials in which the hand and cup orientation were compatible (hand grasping the cup from its lip) were significantly shorter (72 ms; SEM = 14.8 ms) than the ones in which participants grasped the closed end of the cup (*t*_39_ = -2.25, *p* = 0.03). There were significant main effects of ACTION [*F*(1,37) = 23.89, h^2^ = 0.39, *p* < 0.0001] as MTs for lift actions were on average 143 ms (SEM = 24.5 ms) shorter than those for turn actions and END-STATE COMFORT [*F*(1,37) = 35.96, h^2^ = 0.49, *p* < 0.0001], indicating that MTs for actions that ended comfortably were on average 99.55 ms (SEM = 16.9 ms) shorted than those for actions that ended uncomfortably.

There were significant two-way interactions of COMPATIBILTY by GROUP [*F*(1,37) = 3.39, h^2^ = 0.15, *p* = 0.045] and END-STATE COMFORT by GROUP [*F*(1,37) = 4.44, h^2^ = 0.19, *p* = 0.019]. The ACTION by GROUP interaction was not significant [*F*(1,37) = 2.12, h^2^ = 0.18, *p* = 0.35]. *Post hoc* analyses are shown in the Supplementary Material.

There were significant two-way interactions of ACTION by END-STATE COMFORT [*F*(1,37) = 80.41, h^2^ = 0.685, *p* < 0.0001] and COMPATIBILITY by ACTION [*F*(1,37) = 6.49, h^2^ = 0.15, *p* = 0.015].

The former interaction reflected the difference in end-state comfort advantage on MTs between lift and turn actions. *Post hoc t*-tests revealed that in lift actions, MTs were on average 229 ms (SEM = 12.5 ms) shorter if they ended comfortably (*t*_39_ = -9.8, *p* < 0.0001); whereas in turn actions, there was no significant END-STATE COMFORT effect on MTs (*t*_39_ = 0.8, *p* > 0.1).

The COMPATIBILITY by ACTION interaction arose because there was no effect of compatibility on MTs for lift actions (*t*_39_ = -1.5, *p* > 0.1), whereas there was a significant effect of compatibility on turn actions (*t*_39_ = -3.04, *p* = 0.004).

There were also significant three-way COMPATIBILTY by ACTION by END-STATE COMFORT [*F*(1,37) = 14.18, h^2^ = 0.28, *p* = 0.001] and four-way interactions of GROUP by COMPATIBILTY by ACTION by END-STATE COMFORT [*F*(2,37) = 6.42, h^2^ = 0.26, *p* = 0.004].

**FIGURE 4:**
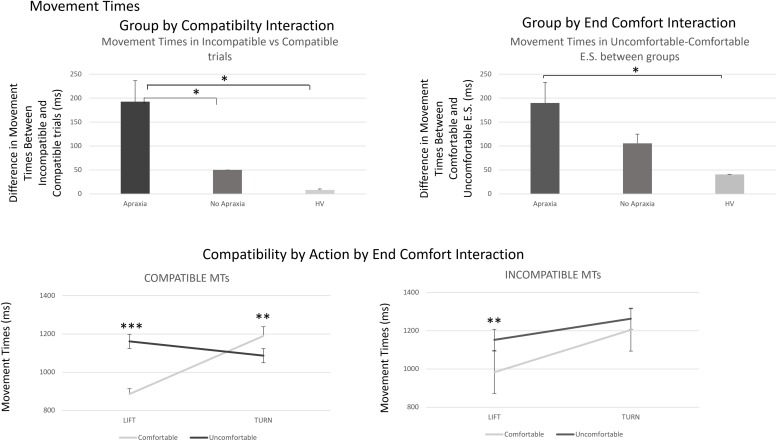
Movement times – interactions by group and three-way interaction of compatibility by action by end-state comfort effects. The **top panel** demonstrates the group by compatibility and end-state comfort effects in MTs, which reflect a similar process to what was described for error rates in Figure 2. The **bottom panel** describes the compatibility by action by end-comfort interaction identified for MTs. This arose because of a significant difference in end-state comfort effects for lift compared to turn actions in compatible trials, which was only present in lift, and not turn, actions, in incompatible trials. The four-way interaction identified that the effect in compatible trials was driven by patients with ideomotor apraxia, indicating an increased sensitivity to end-state comfort effects in compatible trials for these patients. ^∗^*p* < 0.05, ^∗∗^*p* < 0.001, and ^∗∗∗^*p* < 0.0001.

The *post hoc* analyses for these are reported in the Supplementary Material and are shown in Figure 4.

## Discussion

This study investigated factors influencing motor planning and performance when grasping to manipulate a familiar object in healthy volunteers and left hemisphere stroke patients with and without apraxia. We identified that both factors relating to perceptual processing of the object (“compatibility”) and the ones that related to action outcomes, in this case determined by the “end-state comfort” effect, were differentially modulated during task performance in patients with apraxia following a stroke. We identified these differences using measures of motor preparation and performance.

In the sections below, we outline our main findings and discuss these in relation to traditional models of apraxia and brain mechanisms underpinning the disorder.

### Deficits in Integrating Perceptual Knowledge During Goal-Directed Actions in Apraxia

Our study identified differences in performance, measured using error rates and speed of movement, between healthy volunteers and stroke patients with and without apraxia. Error rates and initiation times were more elevated in stroke patients compared to healthy volunteers.

A study by Belanger et al. (1996) demonstrated that, when testing patients on neuropsychological tasks, apraxic deficits related not to the categories of praxis tasks but to task difficulty. This effect was independent of movement type or methods of movement elicitation. Our study supports these findings in that we identified that praxis deficits in our patient cohort significantly correlated with severity of impairment following their stroke, measured on the “ARAT” scale.

However, our results go further than that. We observed that patients with apraxia showed deficits that were specific when they manipulated a common object. This is despite the fact that they showed near-normal performance when grasping objects on apraxia screening tasks.

Stroke patients with and without apraxia made significantly more errors and were slower at initiating actions for trials that ended uncomfortably compared to the ones that ended comfortably, compared to healthy volunteers. This would support a generic deficit in completing actions for which the outcome was difficult or unrewarding, such as when ending in an uncomfortable hand position.

In addition, patients with apraxia made more errors on incompatible trials. There was a significant modulation of the degree of sensitivity of response-outcome (end-state comfort) effects measured during MTs, by compatibility of the initial hand position with the object, and action type (lift or turn). In compatible trials, there were no significant differences in MTs between turn actions that ended comfortably and uncomfortably in healthy volunteers and patients with no apraxia. However, in patients with apraxia, MTs were paradoxically shorter for turns that ended uncomfortably compared to the ones that ended comfortably. This was because they displayed an increased cost in inverting their grasp to reach the cup, despite the action leading to a preferred action outcome. This result suggests that in patients with apraxia, the action goal was trumped by the biomechanical requirement of the turn action, which was easier, in the uncomfortable end-state condition compared to the comfortable one. Moreover, patients with apraxia showed delayed MTs for actions that were not compatible even when they ended comfortably, suggesting an overreliance on compatibility at the expense of the end-state comfort effect in these patients.

### Comparisons of Performance in This Task With Traditional Theories of the Disorder

Traditional theories of apraxia distinguish patients based on deficits they show in neuropsychological tasks. “Ideational” apraxia can be tested by demonstrating deficits in single-object use, or in sequencing errors in multiobject use (Poeck, 1986). “Ideomotor” apraxia can be elicited by asking patients to imitate gestures and by demonstrating spatiotemporal errors (rather than errors of content) when patients are asked to pantomime or use objects (Wheaton and Hallett, 2007). Cognitive models of the disorder conjecture separable underlying mechanisms for these types of deficits: one requires semantic processing of gestures based on knowledge, and the other requires implementation of gestures based on structural–mechanical problem solving. Nevertheless, these subdivisions have failed to fully explain the disorder, with some authors arguing that these represent one and the same problem, leading to the use of inconsistent terminology (Buxbaum, 2001; Hanna-Pladdy and Rothi, 2001).

As in other studies, we did not find deficits that would be predicted based on this dichotomy. We discuss below how deficits in ideational or ideomotor apraxia would be anticipated to influence our results, and reasons for identifying both types in our task, which we attribute to deficits in incorporating affordances in a “hierarchy of goals” framework (Bekkering et al., 2005).

#### Comparisons of Our Results in Relation to Deficits Pertaining to Ideational Apraxia

Patients with ideational deficits, such as patients with semantic dementia, demonstrate a preserved ability to elicit how an object can be grasped from its structure (Hodges et al., 1999), while having deficits in identifying or eliciting appropriate actions relating to their use (Hodges et al., 2000; Bozeat et al., 2002). These deficits can be viewed as problems in identifying and implementing action “goals.” In multiobject use tasks, these problems could be confounded by an inability to sequence and task difficulty (Belanger et al., 1996; Hanna-Pladdy and Rothi, 2001).

In our task, we would predict that if our patients had ideational deficits, they would show impairments in processing the end-state comfort effect. One possibility would be that patients with a deficit in this task condition might show an inability to differentiate comfortable from uncomfortable end postures, leading to prolonged error rates or movement preparation times in task conditions ending comfortably. Another possibility, which was indeed observed in study, was that stroke patients (with and without apraxia) would overrely on the end-state comfort effect such that patients showed a greater deficit in task conditions that ended uncomfortably than did healthy volunteers. Errors and MTs in these conditions were more elevated in stroke patients with apraxia compared to healthy volunteers. It is interesting that we did observe this deficit in our patient cohort, despite the fact that they did not appear to have ideational deficits on neuropsychological testing (noting that we had not tested them on multiobject use). Deficits relating to ideational apraxia vary in their definition, creating confusion on whether the disorder is truly separable from ideomotor deficits (Hanna-Pladdy and Rothi, 2001). We would argue that our task suggests an element of ideation deficit in our patients, based on original descriptions of this deficit being related to sequencing and complexity (Poeck, 1986).

#### Comparisons of Our Results in Relation to Deficits Pertaining to Ideomotor Apraxia

Ideomotor deficits have been described in the apraxia literature as corresponding to problems in “mechanical problem solving” (Osiurak et al., 2008a; Goldenberg and Spatt, 2009). These lead to deficits in accurately grasping or manipulating an object despite knowing how to use it (Daprati and Sirigu, 2006). Recent studies have demonstrated that patients with ideomotor deficits have problems inferring function from object structure, relating to “affordances” (Barde et al., 2007). Affordances represent a mechanism that triggers actions based on a stimulus–response association, which is dependent on task demands (Balleine and Dickinson, 1998; Bub and Masson, 2010). Affordances can elicit actions that represent movement preference to an object. In the case of a cup, as used in our experiment, this would be related to a compatibility effect between cup and hand orientation when it is grasped. In our study, both healthy volunteers and patients preferred to grasp the cup from its open end.

Studies in patients with apraxia have shown an overreliance of these patients in compatible (or “afforded”) trial conditions. A series of experiments have identified that patients with apraxia show increased deficits when manipulating “conflict” objects, which can elicit different grasp and hand movements, for moving or using them (Jax and Buxbaum, 2013; Watson and Buxbaum, 2014, 2015). Lee et al. (2014) identified that patients with ideomotor apraxia were unable to select actions elicited by object affordances in the presence of distractors. In a similar vein, studies on patients with alien-limb syndrome have shown deficits in performing tasks in which conflicting movements may be elicited by affordances (Riddoch et al., 1998; McBride et al., 2012).

#### Summary of the Conditions Causing Deficits in This Task

Our results were able to identify specific task conditions in which deficits were more likely to occur in apraxic patients compared to patients with no apraxia and healthy volunteers. These involved both deficits in processing compatibility and in end-state comfort. When tested on traditional neuropsychological tasks, our patients exhibited predominantly ideomotor deficits. However, the deficits identified in performance of this task were more complicated to interpret and suggest a combination of more generic (complexity-related) and more specific (affordance- and end-state comfort-related) deficits.

Patients with apraxia had greater difficulty in completing actions that ended uncomfortably. This could possibly suggest a generic deficit in complex task performance (Belanger et al., 1996) or in identifying action goals, both of which have traditionally been attributed to ideational deficits (Hanna-Pladdy and Rothi, 2001).

Conversely, deficits and delays in processing incompatible trial conditions would reflect deficits that have previously been attributed in “mechanical problem solving.” These deficits may reflect an overreliance of apraxic patients on afforded trials, at the expense of other trial conditions (Riddoch et al., 1998; McBride et al., 2012; Lee et al., 2014). This could result from an inability to reprogram an action in relation to its goal instead of its affordance. The other possibility would be that these deficits arise due to a failure to incorporate perceptual features of objects into sequential action goals.

In a study by Bekkering et al. (2005), patients with ideomotor apraxia made imitation errors due to deficits in implementing action goals, hierarchically. Their deficits were present both when targeting body parts and when targeting objects, which the authors described as demonstrating deficits in a “hierarchy of goals” framework (Grafton and Hamilton, 2007). In a similar vein, our patients with ideomotor apraxia were compromised when carrying actions that ended in an uncomfortable end state, compared to other groups. However, they were particularly compromised when performing actions that were incompatible. Habitual actions associate stimuli with responses that were previously rewarding (Herbort and Butz, 2011; Voon et al., 2015). There is evidence that the two (goal-directed and habitual) systems parallel each other (Balleine and Dickinson, 1998; Herbort and Butz, 2011; Dolan and Dayan, 2013). Our results suggest an interference of one system on the other (Chainay and Humphreys, 2002).

Taken together, our results have demonstrated that patients with ideomotor apraxia may have deficits in selecting among competing actions elicited by parallel action systems: a perceptual/habitual and a goal-directed system. These deficits are important in terms of identifying how patients with apraxia may fail in activities of daily living (Bickerton et al., 2012). One method traditionally used for rehabilitation of this disorder (West et al., 2008), namely, errorless learning, could be helpful both in recreating a habitual, stimulus–response, movement repertoire and in enabling this to be more readily incorporated during action sequences.

## Conclusion

In this study, we investigated the performance of patients with ideomotor apraxia on a task that involved manipulating a familiar object, namely, a cup. We compared their performance with patients who did not have apraxia and healthy, age-matched, volunteers. The task required participants to either lift or turn the cup, which led to comfortable or uncomfortable end states. We observed that patients with apraxia were impaired both when actions were incompatible with the familiar way of grasping the object (its “affordance”) and in conditions that ended with an uncomfortable end state, suggesting a deficit in goal-directed actions. Both suggest an increased reliance of patients with ideomotor apraxia on these systems compared to stroke patients without apraxia and healthy volunteers.

Rather than an impairment in representing “affordances,” this study suggests that praxis deficits may affect sensitivity to actions elicited by affordances and goal-directed actions in a dynamic fashion. These system are known to operate in parallel and may be competing at specific times of action implementation (Chainay and Humphreys, 2002; Voon et al., 2015). Further studies investigating how these interact dynamically, using decision-making tasks, would help elucidate the exact deficits observed in these patients.

## Data Availability Statement

The datasets generated for this study are available on request to the corresponding author.

## Ethics Statement

Written informed consent was obtained from the individual(s) for the publication of any potentially identifiable images or data included in this article. The patients and participants provided their written informed consent to participate in this study, which was approved by the National Research Ethics Service (NRES) South Central Berkshire Health Research Authority (14/SC/0074).

## Author Contributions

ER conceptualized the task and study. MD programmed the task on MATLAB. GP carried out the data collection on all participants including patients and healthy volunteers. ER and ZZ analyzed the data and edited the manuscript. ER wrote the manuscript.

## Conflict of Interest

The authors declare that the research was conducted in the absence of any commercial or financial relationships that could be construed as a potential conflict of interest.

## References

[B1] Balleine B. W. Dickinson A. (1998). Goal-directed instrumental action: contingency and incentive learning and their cortical substrates. *Neuropharmacology* 37 407–419. 10.1016/s0028-3908(98)00033-1 9704982

[B2] Barde L. H. Buxbaum L. J. Moll A. D. (2007). Abnormal reliance on object structure in apraxics’ learning of novel object-related actions. *J. Int. Neuropsychol. Soc.* 13 997–1008. 10.1017/s1355617707070981 17942018

[B3] Bekkering H. Brass M. Woschina S. Jacobs A. M. (2005). Goal-directed imitation in patients with ideomotor apraxia. *Cogn. Neuropsychol.* 22 419–432. 10.1080/02643290442000275 21038259

[B4] Belanger S. A. Duffy R. J. Coelho C. A. (1996). The assessment of limb apraxia: an investigation of task effects and their cause. *Brain Cogn.* 32 384–404. 10.1006/brcg.1996.0072 8975678

[B5] Bickerton W. Riddoch M. Samson D. Balani A. Mistry B. Humphreys G. (2012). Systematic assessment of apraxia and functional predictions from the birmingham cognitive screen. *J. Neurol. Neurosurg. Psychiatry* 83 513–521. 10.1136/jnnp-2011-300968 22383734

[B6] Bozeat S. Ralph M. A. Patterson K. Hodges J. R. (2002). The influence of personal familiarity and context on object use in semantic dementia. *Neurocase* 8 127–134. 10.1093/neucas/8.1.127 11997491

[B7] Bub D. N. Masson M. E. J. (2010). Grasping beer mugs: on the dynamics of alignment effects induced by handled objects. *J. Exp. Psychol. Hum. Percept. Perform.* 36 341–358. 10.1037/a0017606 20364923

[B8] Buxbaum L. J. (2001). Ideomotor apraxia: a call to action. *Neurocase* 7 445–458. 10.1093/neucas/7.6.445 11788737

[B9] Buxbaum L. J. Sirigu A. Schwartz M. F. Klatzky R. (2003). Cognitive representations of hand posture in ideomotor apraxia. *Neuropsychologia* 41 1091–1113. 10.1016/s0028-3932(02)00314-7 12667544

[B10] Chainay H. Humphreys G. W. (2002). Privileged access to action for objects relative to words. *Psychon. Bull. Rev.* 9 348–355. 10.3758/bf03196292 12120799

[B11] Cisek P. (2005). Neural representations of motor plans, desired trajectories, and controlled objects. *Cogn. Process* 6 15–24. 10.1007/s10339-004-0046-7

[B12] Creem S. H. Proffitt D. R. (2001). Grasping objects by their handles: a necessary interaction between cognition and action. *J. Exp. Psychol. Hum. Percept. Perform.* 27 218–228. 10.1037/0096-1523.27.1.218 11248935

[B13] Daprati E. Sirigu A. (2006). How we interact with objects: learning from brain lesions. *Trends Cogn. Sci.* 10 265–270. 10.1016/j.tics.2006.04.005 16678468

[B14] Dolan R. J. Dayan P. (2013). Goals and habits in the brain. *Neuron* 80 312–325. 10.1016/j.neuron.2013.09.007 24139036 PMC3807793

[B15] Ellis R. Tucker M. (2000). Micro-affordance: the potentiation of components of action by seen objects. *Br. J. Psychol.* 91(Pt 4), 451–471. 10.1348/000712600161934 11104173

[B16] Gibson J. J. (1979). *The Ecological Approach to Visual Perception.* Boston: Houghton Mifflin.

[B17] Goldenberg G. Spatt J. (2009). The neural basis of tool use. *Brain* 132 1645–1655. 10.1093/brain/awp080 19351777

[B18] Grafton S. T. Hamilton A. F. (2007). Evidence for a distributed hierarchy of action representation in the brain. *Hum. Mov. Sci.* 26 590–616. 10.1016/j.humov.2007.05.009 17706312 PMC2042582

[B19] Hanna-Pladdy B. Rothi L. J. G. (2001). Ideational apraxia: confusion that began with Liepmann. *Neuropsychol. Rehabil.* 11 539–547. 10.1080/09602010143000022

[B20] Harris C. M. Wolpert D. M. (1998). Signal-dependent noise determines motor planning. *Nature* 394 780–784. 10.1038/29528 9723616

[B21] Herbort O. Butz M. V. (2011). Habitual and goal-directed factors in (everyday) object handling. *Exp. Brain Res.* 213 371–382. 10.1007/s00221-011-2787-8 21748333

[B22] Herbort O. Butz M. V. (2015). Planning grasps for object manipulation: integrating internal preferences and external constraints. *Cogn. Process* 16(Suppl. 1), 249–253. 10.1007/s10339-015-0703-z 26224266

[B23] Herbort O. Mathew H. Kunde W. (2017). Habit outweighs planning in grasp selection for object manipulation. *Cogn. Psychol.* 92 127–140. 10.1016/j.cogpsych.2016.11.008 27951435

[B24] Hodges J. R. Bozeat S. Lambon Ralph M. A. Patterson K. Spatt J. (2000). The role of conceptual knowledge in object use evidence from semantic dementia. *Brain* 123(Pt 9), 1913–1925. 10.1093/brain/123.9.1913 10960055

[B25] Hodges J. R. Spatt J. Patterson K. (1999). “What” and “how”: evidence for the dissociation of object knowledge and mechanical problem-solving skills in the human brain. *Proc. Natl. Acad. Sci. U.S.A.* 96 9444–9448. 10.1073/pnas.96.16.9444 10430962 PMC17802

[B26] Humphreys G. W. Bickerton W. L. Samson D. Riddoch M. J. (2012). *BCoS Cognitive Screen.* London: Psychology Press.

[B27] Jax S. A. Buxbaum L. J. (2013). Response interference between functional and structural object-related actions is increased in patients with ideomotor apraxia. *J. Neuropsychol.* 7 12–18. 10.1111/j.1748-6653.2012.02031.x 22515637 PMC4019205

[B28] Jeannerod M. (1994). The representing brain: Neural correlates of motor intention and imagery. *Behav. Brain Sci.* 17 187–202. 10.1017/S0140525X00034026

[B29] Keele S. W. (1968). Movement control in skilled motor performance. *Psychol. Bull.* 70(6, Pt.1), 387–403. 10.1037/h0026739

[B30] Lee C. I. Mirman D. Buxbaum L. J. (2014). Abnormal dynamics of activation of object use information in apraxia: evidence from eyetracking. *Neuropsychologia* 59 13–26. 10.1016/j.neuropsychologia.2014.04.004 24746946 PMC4096147

[B31] Lyle R. C. (1981). A performance test for assessment of upper limb function in physical rehabilitation treatment and research. *Int. J. Rehabil. Res.* 4 483–492. 10.1097/00004356-198112000-00001 7333761

[B32] Marteniuk R. G. MacKenzie C. L. Jeannerod M. Athenes S. Dugas C. (1987). Constraints on human arm movement trajectories. *Can. J. Psychol.* 41 365–378. 10.1037/h0084157 3502905

[B33] McBride J. Sumner P. Husain M. (2012). Conflict in object affordance revealed by grip force. *Q. J. Exp. Psychol.* 65 13–24. 10.1080/17470218.2011.588336 21824035 PMC3259623

[B34] Oldfield R. C. (1971). The assessment and analysis of handedness: the Edinburgh inventory. *Neuropsychologia* 9 97–113. 10.1016/0028-3932(71)90067-4 5146491

[B35] Osiurak F. Aubin G. Allain P. Jarry C. Etcharry-Bouyx F. Richard I. (2008a). Different constraints on grip selection in brain-damaged patients: object use versus object transport. *Neuropsychologia* 46 2431–2434. 10.1016/j.neuropsychologia.2008.03.018 18462765

[B36] Osiurak F. Aubin G. Allain P. Jarry C. Richard I. Le Gall D. (2008b). Object utilization and object usage: a single-case study. *Neurocase* 14 169–183. 10.1080/13554790802108372 18569742

[B37] Osterrieth P. A. (1944). Test of copying a complex figure; contribution to the study of perception and memory. *Arch. Psychol.* 30 206–356. 17007938

[B38] Poeck K. (1986). The clinical examination for motor apraxia. *Neuropsychologia* 24 129–134. 10.1016/0028-3932(86)90046-1 3703231

[B39] Rey A. (1941). The psychological examination in cases of traumatic encepholopathy. *Arch. Psychol.* 28 215–285.

[B40] Riddoch M. J. Edwards M. G. Humphreys G. W. West R. Heafield T. (1998). Visual affordances direct action: neuropsychological evidence from manual interference. *Cogn. Neuropsychol.* 15 645–683. 10.1080/026432998381041 22448840

[B41] Riddoch M. J. Humphreys G. W. Price C. J. (1989). Routes to action: Evidence from apraxia. *Cogn. Neuropsychol.* 6 437–454. 10.1080/02643298908253424

[B42] Rinne P. Hassan M. Fernandes C. Han E. Hennessy E. Waldman A. (2018). Motor dexterity and strength depend upon integrity of the attention-control system. *Proc. Natl. Acad. Sci. U.S.A.* 115 536–545. 10.1073/pnas.1715617115 29284747 PMC5776987

[B43] Rosenbaum D. A. Cohen R. G. Meulenbroek R. G. J. Vaughan J. (2006). “Plans for Grasping Objects,” in *Motor Control and Learning*, eds Latash M. L. Lestienne F. (Boston, MA: Springer), 9–25. 10.1007/0-387-28287-4_2

[B44] Rosenbaum D. A. Marchak F. Barnes H. J. Vaughan J. Slotta J. D. Jorgensen M. J. (1990). “Constraints for action selection: Overhand versus underhand grips,” in *Attention and Performance 13: Motor Representation and Control*, ed. Jeannerod M. (Hillsdale, NJ: Lawrence Erlbaum Associates, Inc), 321–342. 10.4324/9780203772010-10

[B45] Rosenbaum D. A. Vaughan J. Barnes H. J. Jorgensen M. J. (1992). Time course of movement planning: selection of handgrips for object manipulation. *J. Exp. Psychol. Learn. Mem. Cogn.* 18 1058–1073. 10.1037/0278-7393.18.5.1058 1402710

[B46] Rounis E. Humphreys G. (2015). Limb apraxia and the “affordance competition hypothesis”. *Front. Hum. Neurosci.* 9:429. 10.3389/fnhum.2015.00429 26283948 PMC4516886

[B47] Rounis E. Yarrow K. Rothwell J. C. (2007). Effects of rTMS conditioning over the fronto-parietal network on motor versus visual attention. *J. Cogn. Neurosci.* 19 513–524. 10.1162/jocn.2007.19.3.513 17335398

[B48] Rounis E. Zhang Z. Pizzamiglio G. Duta M. Humphreys G. W. (2017). Factors influencing planning of a familiar grasp to an object: what it is to pick a cup. *Exp. Brain Res.* 235 1281–1296. 10.1007/s00221-017-4883-x 28204861 PMC5348548

[B49] Rushworth M. F. Ellison A. Walsh V. (2001). Complementary localization and lateralization of orienting and motor attention. *Nat. Neurosci.* 4 656–661. 10.1038/88492 11369949

[B50] Sirigu A. Cohen L. Duhamel J. R. Pillon B. Dubois B. Agid Y. (1995). A selective impairment of hand posture for object utilization in apraxia. *Cortex* 31 41–55. 10.1016/s0010-9452(13)80104-9 7781320

[B51] Tucker M. Ellis R. (1998). On the relations between seen objects and components of potential actions. *J. Exp. Psychol. Hum. Percept. Perform.* 24 830–846. 10.1037//0096-1523.24.3.830 9627419

[B52] Ulrich R. Miller J. (1994). Effects of truncation on reaction time analysis. *J. Exp. Psychol. Gen.* 123 34–80. 10.1037//0096-3445.123.1.34 8138779

[B53] Voon V. Baek K. Enander J. Worbe Y. Morris L. S. Harrison N. A. (2015). Motivation and value influences in the relative balance of goal-directed and habitual behaviours in obsessive-compulsive disorder. *Transl. Psychiatry* 5:e670. 10.1038/tp.2015.165 26529423 PMC5068758

[B54] Watson C. E. Buxbaum L. J. (2014). Uncovering the architecture of action semantics. *J. Exp. Psychol. Hum. Percept. Perform.* 40 1832–1848. 10.1037/a0037449 25045905 PMC4224273

[B55] Watson C. E. Buxbaum L. J. (2015). A distributed network critical for selecting among tool-directed actions. *Cortex* 65 65–82. 10.1016/j.cortex.2015.01.007 25681649 PMC4385438

[B56] Welford A. T. (1968). *Fundamentals of Skill.* London: Methuen.

[B57] West C. Bowen A. Hesketh A. Vail A. (2008). Interventions for Motor Apraxia Following Stroke. *Cochrane Database Syst. Rev.* 2008:CD004132. 10.1002/14651858.CD004132.pub2 PMC6464830 18254038

[B58] Wheaton L. A. Hallett M. (2007). Idemotor apraxia: a review. *J. Neurol. Sci.* 260 1–10. 17507030 10.1016/j.jns.2007.04.014

[B59] Wolpert D. M. Miall R. C. (1996). Forward models for physiological motor control. *Neural Netw.* 9 1265–1279. 10.1016/s0893-6080(96)00035-4 12662535

[B60] Wong A. L. Haith A. M. Krakauer J. W. (2015). Motor planning. *Neuroscientist* 21 385–398. 10.1177/1073858414541484 24981338

