# Supplementary material for: Factors Influencing Manipulation of a Familiar Object in Patients With Limb Apraxia After Stroke
Source: Front Hum Neurosci. 2020 Feb 11;13:465. doi: 10.3389/fnhum.2019.00465 (PMC7026485; doi:10.3389/fnhum.2019.00465)
Supplement: Supplementary file 1 [file Table_1.DOCX]

**Factors influencing manipulation of a familiar object in patients with limb apraxia after stroke**

**Results:**

*Post-Hoc analyses of Results:*

1. *Error Rates*

This analysis revealed significant GROUP-by-COMPATIBILITY [F(1,2)=4.35, η^2^=0.19, p=0.02] and GROUP-by-END-STATE COMFORT [F(1,2)=4.28, η^2^=0.19, p=0.02]) interactions.

The GROUP-by-COMPATABILTY interaction was further examined by post hoc two-sample t-tests (assuming unequal variances) comparing the compatibility effects on errors, between each pair of groups. The compatibility effect was significantly larger for patients with apraxia than both patients without apraxia (t_11_=2.81, p=0.03) and healthy controls (t_9_=2.4, p=0.04), while the compatibility effect was not significantly different between patients without apraxia and healthy controls (t_16_=0.7, p=0.47). Figure 2 shows the error rate differences in relation to compatibility for each group.

Post-hoc t-tests were carried out to test the GROUP by END-STATE COMFORT interaction, comparing the END-STATE COMFORT effect between each pair of groups. The END-STATE COMFORT effect was significantly larger for both patients with and without apraxia, compared to healthy controls (patients with no apraxia versus controls t_11_=-2.32, p=0.04; patients with apraxia versus controls t_9_=-2.5, p=0.03), while there was no significant difference between the apraxia and no apraxia groups (t_19_=0.59, p=0.56). These results are shown in Figure 2.

There was a trend for a 2-way interaction of COMPATIBILITY BY ACTION [F(1, 37)=3.72, η^2^=0.09, p=0.06], and a significant 3-way GROUP by COMPATIBILITY BY ACTION interaction [F(2, 37)=3.4, η^2^=0.15, p=0.044]. We ran post-hoc ANOVAs of COMPATIBILITY BY ACTION for each of the participant groups. The interaction between these two factors was not significant in any of the participant groups (healthy volunteers, [F(1, 17)=2.7, η^2^=0.14, p=0.11], patients with apraxia [F(1, 9)=2.5, η^2^=0.22, p=0.15], patients without apraxia [F(1, 17)=3.7, η^2^=0.23, p=0.07], so no further analyses were carried out.

The remaining two-, three- and four-way interactions were not significant.

1. *Initiation Times:*

To investigate the GROUP by END-STATE COMFORT interaction on initiation times, we examined the END-STATE COMFORT effects, comparing them between each of the two groups. We found that in stroke patients with apraxia and healthy volunteers, END-STATE COMFORT effects were significant, with initiation times for actions that ended comfortably being significantly shorter (Apraxia: 141.6ms, t_9_=-2.78, p=0.02; healthy volunteers 36.99ms, t_17_=-2.98, p=0.008). Conversely in stroke patients without apraxia, the END-STATE COMFORT effect was not significant (t_11_=-1.36, p=0.2). Between group comparisons showed that the END-STATE COMFORT effect was significantly larger for patients with apraxia than both patients without apraxia (t_14_=-2.86 , p=0.043) and healthy controls (t_10_=-2.99, p=0.037), while the difference between patients without apraxia and healthy controls was not significant (t_16_=0.01, p=0.99) (Figure 3, left panel).

Post-hoc t-tests for the ACTION by END-STATE COMFORT interaction revealed that the END-STATE COMFORT effect was significant for the lift action (t_39_=-3.1, p=0.003), but not significant for the turn action (t_39_=-1.06, p=0.3) (Figure, 3, right panel).

The remaining 2-, 3- and 4-way interactions were not significant.

The results for initiation times is shown in Figure 3.

1. *Movement Times:*

Post-hoc t-tests comparing the effects of COMPATIBILITY in each GROUP revealed that the COMPATIBLITY effect on MTs was significant for patients with apraxia only (with compatible trials 192ms faster, t_9_=-2.8, p=0.017), but not significant for other two groups (p>0.1). Between-group comparisons showed that the COMPATIBILITY effect on MTs was significantly larger for patients with apraxia than the other two groups (patients with no apraxia t_9_=3.25, p=0.024; healthy volunteers t_9_=3.02, p=0.03), while it was not significantly different between patients without apraxia and health controls (t_18_=1.43, p=0.14).

Post hoc t-tests for the effect of GROUP by END-STATE COMFORT interaction revealed that while the END-STATE COMFORT effect was significant in all of the 3 groups (patients with apraxia, (t_9_=-3.64, p=0.005); patients without apraxia, (t_11_=-3.02, p=0.012); healthy controls, (t_11_=-3.85, p=0.001), it was significantly larger for patients with apraxia compared with healthy volunteers (t_10_=2.78, p=0.02), but the other two between-group comparisons were not significant (p>0.1). These results are depicted in Figure 4 (top panel).

The ACTION by END-STATE COMFORT interaction reflected the difference in End-state comfort advantage on MTs between lift and turn actions. Post hoc t-tests revealed that in lift actions, MTs were on average 229ms (SEM=12.5ms) shorter if they ended comfortably (t_39_=-9.8, p<0.0001); whereas in turn actions, there was no significant END-STATE COMFORT effect on MTs (t_39_=0.8, p>0.1).

The COMPATIBILITY by ACTION interaction arose because there was no effect of compatibility on MTs for lift actions (t_39_=-1.5, p>0.1), whereas there was a significant effect of compatibility on turn actions (t_39_=-3.04, p=0.004).

The three-way COMPATIBILTY by ACTION by END-STATE COMFORT interaction was further explored by examining the two-way ACTION by END-STATE COMFORT interactions under compatible and incompatible conditions, respectively. The ANOVA for COMPATIBLE trials revealed significant main effects of ACTION [F(1,39)=15.77, η^2^=0.29, p<0.0001] and END-STATE COMFORT [F(1,39)=14.84, η^2^=0.28, p<0.0001], and a significant interaction between the two F(1,38)=34.93, η^2^=0.48, p<0.0001]. The ANOVA for INCOMPATIBLE trials, also revealed significant main effects of ACTION [F(1,39)=16.62, η^2^=0.299, p<0.0001], END-STATE COMFORT [F(1,39)=21.25, η^2^=0.35, p<0.0001] but there was no significant interaction between the two [F(1,39)=2.88, η^2^=0.09, p=0.083].

Post-hoc t-tests revealed that in *compatible* trials, MTs for lift actions which started and ended comfortably were shorter on average than ones that started and ended uncomfortably (t_39_=9.96, p<0.0001), indicating a cost for reaching with a hand pronation at the start in the latter condition. MTs for turn actions were significantly shorter for a cup turned from upright to down (leading to an uncomfortable end state) compared to turning a cup from down to up (leading to a comfortable end state) (difference of 103.3ms SEM 3.3ms; t_39_=3.66, p=0.0007). *This finding represents a violation of the End-state comfort effect in favour of the compatibility (ie. compatibility trumps End-state comfort).* In *incompatible* trials MTs for lift actions were shorter if they started and ended comfortably than uncomfortably (168.6ms, SEM 7.6ms; t_39_=-7.6, p<0.0001). MTs for incompatible turns were no different whether they started comfortably and ended uncomfortably or vice versa (t_39_=-1.3, p=0.2). These results are shown in Figure 4.

Finally, the four-way GROUP-by-COMPATIBILITY-by-ACTION-by-END-STATE COMFORT interaction was explored by entering MTs in three-way ANOVAS and checking the COMPATIBILITY-by-ACTION-by-END-STATE COMFORT interaction for each of the participant groups. This 3-way interaction was significant for patients with apraxia [F(1,9)=6.26, η^2^=0.41, p=0.034], but not for patients without apraxia [F(1,11)=3.33, η^2^=0.232, p=0.09] or for healthy volunteers [F(1,17)=2.46, η^2^=0.13, p=0.13]. For simplicity we describe the relevant interaction for this effect alone: this occurred because, in patients with apraxia, there was no significant effect of End-state comfort on MTs for turn actions in compatible trials (t_9_=0.8, p=0.44). Conversely, MTs for turn actions in incompatible trials were significantly shorter (182.9ms, SEM = 13.4ms) when they ended uncomfortably, than when they ended comfortably (t_9_=-3.6, p=0.0057). This indicating a preference for the cup to be turned upright, compared to down, in the condition where the cup was picked up from its bottom end (incompatible trials).
